# Review of MEMS Based Fourier Transform Spectrometers

**DOI:** 10.3390/mi11020214

**Published:** 2020-02-20

**Authors:** Junyu Chai, Kun Zhang, Yuan Xue, Wenguang Liu, Tian Chen, Yao Lu, Guomin Zhao

**Affiliations:** 1 College of Advanced Interdisciplinary Studies, National University of Defense Technology, Changsha 410073, China; junyuchai@nudt.edu.cn (J.C.); cht_eve@163.com (T.C.); luyao14@nudt.edu.cn (Y.L.); gmzhao@nudt.edu.cn (G.Z.); 2 State Key Laboratory of Pulsed Power Laser Technology, Changsha 410073, China; 3 Hunan Provincial Key Laboratory of High Energy Laser Technology, Changsha 410073, China; 4 College of Computer Science, National University of Defense Technology, Changsha 410073, China; kun.zhang.cc@foxmail.com; 5 Wuxi WiO Technologies Co., Ltd., Wuxi 214000, China; yxue@wiotek.com

**Keywords:** Fourier transform spectrometers, Fourier transform infrared spectrometers (FTIR), micro-electromechanical system, micromirror, microactuator

## Abstract

Fourier transform spectrometers (FTS), mostly working in infrared (IR) or near infrared (NIR) range, provide a variety of chemical or material analysis with high sensitivity and accuracy and are widely used in public safety, environmental monitoring and national border security, such as explosive detection. However, because of being bulky and expensive, they are usually used in test centers and research laboratories. Miniaturized FTS have been developed rapidly in recent years, due to the increasing demands. Using micro-electromechanical system (MEMS) micromirrors to replace the movable mirror in a conventional FTS system becomes a new realm. This paper first introduces the principles and common applications of conventional FTS, and then reviews various MEMS based FTS devices.

## 1. Introduction

Fourier transform infrared spectrometers (FTIR, or more generally FTS) are a type of powerful instruments for chemical and biological sensing. An FTS obtains spectrograms through the Fourier transform of the interferograms generated by an interferometer (Michelson interferometer and lamellar grating interferometer as the two main types) [1]. The spectral ranges of FTS systems can extend from visible to NIR or even MIR. Compared to other spectrometers, such as those based on Fabry-Perot interferometers or gratings, FTS have unparalleled advantages: (1) Multiplexing, so called Fellgett advantage [2], i.e., using only one single photodetector can acquire the full spectral range spectra without the need of any dispersive components and photodetector arrays, greatly reducing the product’s cost and form factor; (2) high throughput, namely Jacquinot advantage [3], resulting from the fact that no slits in the system lead to a higher signal-to-noise ratio (SNR); and (3) linearity, namely Connes advantage [4], so that stable gas lasers, such as a He-Ne laser can be introduced as a reference light source to tackle the interference distortion.

Currently, FTS are no longer restricted to laboratory applications. Microelectromechanical systems (MEMS) technology has been successfully employed to make FTS portable, inexpensive and miniaturized [5,6], so these portable FTS devices can be used for real-time, on-site measurement and analysis. The key to realizing miniature FTS is to replace the bulky scanning mirror module in a conventional FTS with a MEMS mirror. Various MEMS based FTS devices have been developed using electrostatic, electromagnetic and electrothermal microactuators [7,8,9].

This paper is organized as follows. The working principles and applications of conventional FTS are reviewed in Section 2. Several miniaturized FTS utilizing various MEMS based actuation mechanisms are introduced in Section 3.

## 2. Theoretical Background of Fourier Transform Spectrometers 

### 2.1. Fourier Transform Spectroscopy and Its Applications

Fourier transform infrared (IR) spectroscopy reflects the transitions of electrons among the vibrational energy states upon absorbing light [4]. The absorption occurs when an incoming photon excites a molecule to a higher energy state. The transitions between the vibrational states of the molecule occur in the IR range. The wavelength of each IR absorbance peak is dependent on the specific physical and chemical properties of the corresponding molecule, which is a fingerprint of that functional group [10].

Fourier transform spectroscopy analysis has benefits of rapid analysis, minimum sample preparation, noninvasive and nondestructive operation, no reagents needed, simultaneous multicomponent analysis, and compatibility with fiber optics. FTS can also be used to test and characterize optical components, such as light sources, filters, detectors, and fiber-optic components [11]. FTS has been widely used in quality and process control, color measurement, and chemical analysis [4,12]. Compact FTS systems are needed in various fields, such as environmental monitoring, food industry, medical diagnostics, life science, and telecommunication [13,14,15].

### 2.2. Working Principles

#### 2.2.1. Michelson Interferometer-Based FTS

**Figure 1:**
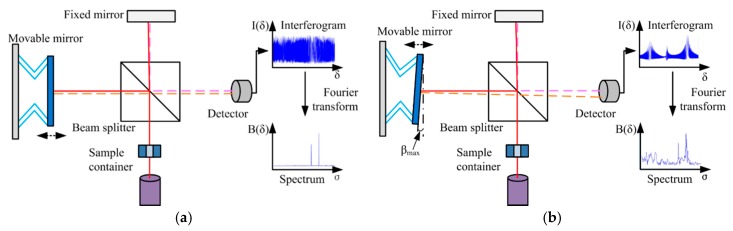
Schematic of a Michelson interferometer: (**a**) An ideal Michelson interferometer; (**b**) a Michelson interferometer with the movable mirror tilting.

As the core of commonly-used FTS systems, Michelson interferometers (see Figure 1a) are typically employed to obtain Fourier Transform spectra [16]. As shown in Figure 1a, the sample light is split by a beam splitter into two light beams: One is directed into a fixed mirror, while the other is incident to a movable mirror. The two reflected light beams recombine at the beam splitter and generate interference signals that are detected by a photodetector. The interferogram signal picked up by the photodetector varies with the piston movement of the movable mirror.

When the sample absorbs a specific wavelength, the interference signal *I*(*δ*) can be expressed as,

(1) $$I\left(\delta \right)=\int_{-\infty }^{+\infty }B\left(\upsilon \right)\cos(2\pi \delta \upsilon )d\upsilon$$

where *δ* is the optical path difference (OPD), *υ* is the wave number, and *B*(*υ*) is the spectral power density. *B*(*υ*) can be obtained by performing Fourier transform on *I*(*δ*), as shown in Equation (2):

(2) $$B\left(\upsilon \right)=\int_{-\infty }^{+\infty }I\left(\delta \right)e^{-i2\pi \delta }d\delta$$

For the FTS system, the theoretical spectral resolution, ∆*σ*, is given by Equation (3):

(3) $$\Delta \sigma =\frac{1}{2\times \Delta Z_{max}}=\frac{1}{\delta_{max}}$$

where Δ*Z_max_* is the maximum displacement of the movable mirror. As shown in Equation (3), the spectral resolution of the FTS is determined by the displacement of the movable micromirror. The resolution increases as the displacement extends. On the other hand, the movable mirror requires to remain in good alignment during the motion. However, in practice, it is difficult to achieve because tilting always exists when the mirror is moving. The tilting of the mirror significantly deteriorates the interferogram (see Figure 1b), thus, causing a reduction of the usable displacement range and degradation of spectral resolution [4]. Therefore, the maximum tilt angle, *β_max_*, without degrading the resolution needs to satisfy the following relationship [17]:

(4) $$\beta_{max}<\frac{1}{20\cdot D\cdot \nu_{max}}$$

where *D* is the diameter of the beam and *ν_max_* is the wavenumber of the shortest wavelength component of the light source. For example, if *D* is 0.1 cm and *ν_max_* is 15,800 cm^−1^, a tilting angle must be less than 0.002°. Hence, the extension of the travel range and the compensation of the tilting are two key factors that can improve the spectral resolution.

#### 2.2.2. Lamellar Grating Interferometer-Based FTS

**Figure 2:**
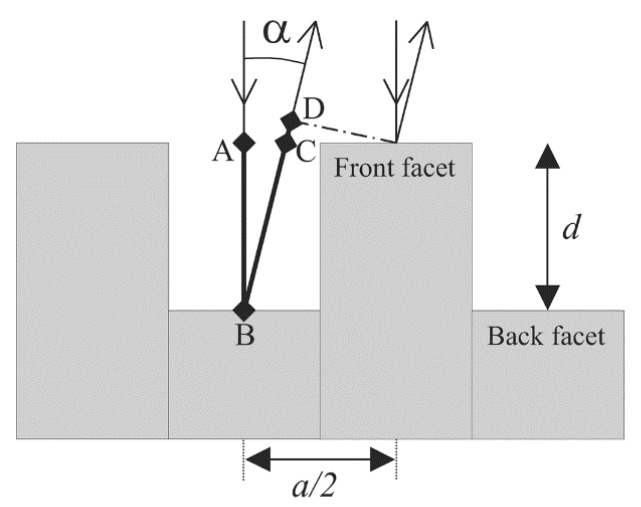
Schematic of a lamellar grating interferometer. (Reprinted with permission from Reference [19] The Optical Society).

A lamellar grating interferometer, which is basically a binary grating with a variable depth that operates in the zeroth order of the diffraction pattern, is also applied as a FTS [18]. Compared with a Michelson interferometer, which splits the wave amplitude via a beam splitter, a lamellar grating interferometer divides the wave front. The lamellar grating (see Figure 2) is made of two sets of facets: One fixed, and the other actuated to move up and down, producing an OPD between two coherent light beams which are reflected off the top surfaces of the two sets of facets. The intensity *I* of the diffraction pattern is given by Reference [19]

(5) $$I\propto {\left(\frac{\sin K}{K}\right)}^{2}{\left(\frac{\sin2nK}{\sin2K}\right)}^{2}\cos^{2}\left(\frac{\varphi }{2}\right)$$

where *K* = (*πa*) *sinα*/(*2λ*), *λ* is the incident wavelength, *a* is the grating period, and *n* is the number of illuminated periods. The diffraction angle *α* and the phase difference *φ* are described as follows:

(6) $$\alpha =\arcsin\left(\frac{m\lambda }{a}\right),\;\varphi =\frac{2\pi }{\lambda }\delta ,$$

where *m* is the diffraction order, and *δ* is the OPD, given by *δ = d* (1 *+ cosα + asinα/*(*2d*)), which is the sum of distances AB, BC, and CD in Figure 2 [19]. When *m* = 0 and *α* = 0, the intensity *I*(*δ*) of the zeroth diffraction order at different OPDs is picked to retrieve the spectrum using Equation (2).

## 3. Fourier Transform Spectrometers Based on MEMS Mirrors

Using MEMS micromirrors to replace the movable mirror modules in conventional Michelson interferometer-based FTS systems is a common solution for miniaturizing FTS. MEMS micromirrors are driven by microactuators. Currently, there are mainly three types of MEMS micromirrors, namely, electrostatic, electromagnetic and electrothermal, that have been used for FTS miniaturization. Another type of micromirrors driven by piezoelectric actuation has also been developed [20,21], but not applied for FTS miniaturization so far, due to the fact that piezoelectric MEMS mirrors tend to have limited piston motion [22,23].

According to the requirements of chemical analysis applications, the resolutions of MEMS based FTS must be 100 cm^−1^ or better [24]. Equation (3) yields that the needed OPD has to be over 100 μm, thus, corresponding the movable mirror needs to travel more than 50 μm, which is a big challenge for MEMS microactuators. On the other hand, the form factor of a MEMS based FTS is also crucial, which directly affects the application range. Therefore, the miniaturization of MEMS based FTS is currently focused on increasing the travel range of the micromirror and reducing its overall size.

### 3.1. Electrostatic MEMS Based FTS

Electrostatic actuation, widely used in the MEMS world for its high speed, low power consumption and easy fabrication, is realized by applying a varying potential difference in the device structure to produce a changing electrostatic force. For example, the digital micromirror device (DMD) (Texas Instruments) that have been successfully commercialized in projection displays is based on electrostatic actuation [25,26]. 

The essence of an electrostatic actuator is a capacitor, which is broadly defined as two conductors that can hold the same or opposite charges. When the distance and the relative position between two conductors change via stimulus, the capacitance value will change accordingly. When a voltage is applied across two conductors, an electrostatic force will be generated between them [27].

**Figure 3:**
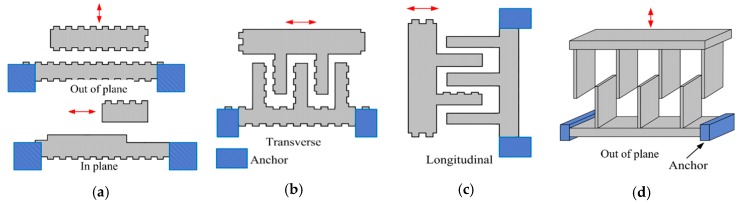
Various configurations of electrostatic actuation: (**a**) Parallel-plate structure (out-of-plane motion and in-plane motion); (**b**) transverse motion comb structure; (**c**) longitudinal motion comb structure; (**d**) out-of-plane motion comb structure.

Parallel-plate and interdigitated fingers (comb drives) are two types of electrostatic actuators available to generate in-plane motions and out-of-plane motions. Two electrodes are involved in each type of electrostatic actuators: One is fixed on the substrate, and the other is suspended and free to move in the desired direction. The parallel-plate structure (see Figure 3a) has planar electrodes facing each other, and the two parallel plates can move with respect to each other in two ways: Normal displacement or parallel sliding displacement [27]. Parallel-plate actuators were applied to form MEMS FTS by VTT [28], where multiple reflections between the two parallel mirror plates were used to increase the OPD (13 reflections at 45° angle, producing a 275 μm OPD). However, the mirror surface had a curvature of up to 200 nm, leading to serious wavefront distortion as the wavefront distortion was amplified upon every reflection. Moreover, the reflection loss would be quite high, due to the multiple reflections. Hence, comb-drive electrostatic actuators are more commonly applied in FTS.

A comb drive consists of two sets of electrodes placed in the same plane parallel to the substrate, which is usually actuated via three operation modes: Transverse, longitudinal and out-of-plane (see Figure 3b–d. (1) Transverse comb drives: The set of free fingers moves in a perpendicular direction to the longitudinal axis of comb fingers; (2) longitudinal comb drives: The direction of relative movement is along the longitudinal axis of the fingers, allowable by the suspension; and (3) Comb drives designed to produce out-of-plane displacement: They are initially located at the same plane and actuated by the fringe capacitance fields. The comb drives, which are commonly used in MEMS FTS, have large length-to-width aspect ratio and rectangular shape viewed from the top and side. The amplitude of the displacement of a comb drive under DC or quasi-static biasing is rather limited. Large piston displacement can be generated from resonant actuation. Several in-plane and out-of-plane electrostatic comb drive MEMS micromirrors and FTS are introduced below.

#### 3.1.1. In-Plane Electrostatic MEMS Micromirrors and FTS

**Figure 4:**
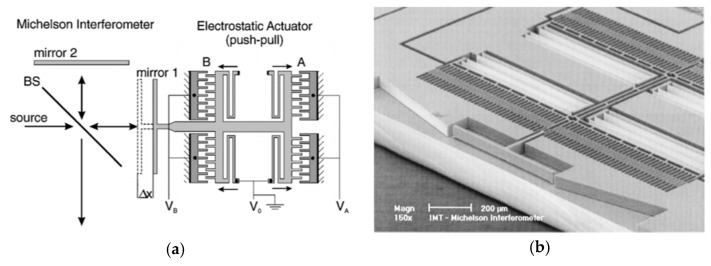
An in-plane electrostatic comb micro-electromechanical system (MEMS) based Fourier transform spectrometers (FTS): (**a**) Schematic of FTS; (**b**) scanning electron microscope (SEM) of the MEMS mirror. (Reprinted with permission from Reference [7] The Optical Society).

A MEMS micromirror driven by an in-plane electrostatic comb was successfully verified in FTS in 1999 by Manzardo et al. [7]. They developed a voltage-movable MEMS mirror to replace the moving mirror in a Michelson interferometer (see Figure 4a). In a conventional comb actuator, the voltage-induced force relied on the square of the control voltage. To overcome this, two identical comb-drive A and B were placed opposite to each other, which could generate a linear movement with variable voltage. The mirror surface was formed by silicon sidewall via deep reactive ion etching (DRIE) (see Figure 4b). When applying a 10 V-amplitude control voltage, the mirror could generate a maximum vertical placement of 77 μm, leading to an OPD of 154 µm. The estimated spectral resolution for He-Ne laser was 5.2 nm. Repeatability of ± 25 nm for the MEMS mirror position from the OPD was measured. However, the mirror size was only about 75 μm × 500 μm, which was much smaller than the size of the entire MEMS device (5 mm × 4 mm). Since the device was based on silicon-on-insulator (SOI) wafer processing, the sidewall mirror surface height was limited by the device layer thickness of the SOI wafer, and thus, greatly limited the FTS system’s luminous flux.

**Figure 5:**
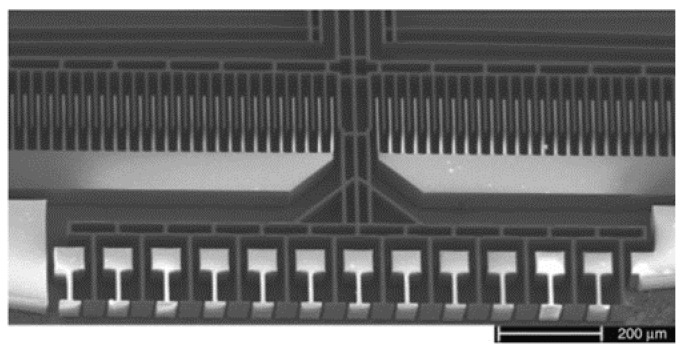
SEM of an in-plane electrostatic actuated Lamellar grating interferometer. (Reprinted with permission from Reference [19] The Optical Society).

An in-plane electrostatically-actuated Lamellar grating interferometer was also proposed in 2004 by Manzardo et al. (see Figure 5) [19]. The actuator was fabricated using DRIE on an SOI wafer. The height of the mirrors was 75 μm, and the number of the illustrated periods of the grating was 12, where the grating period was 90 μm. The whole silicon chip measures at 5 mm × 5 mm. In the experiment, a maximum displacement of 145 μm was achieved with 65 V control voltage, corresponding to a theoretical resolution of 70 cm^−1^. The wavelength range extending from 380 nm to 1100 nm, due to no reflection coating on the silicon mirrors.

**Figure 6:**
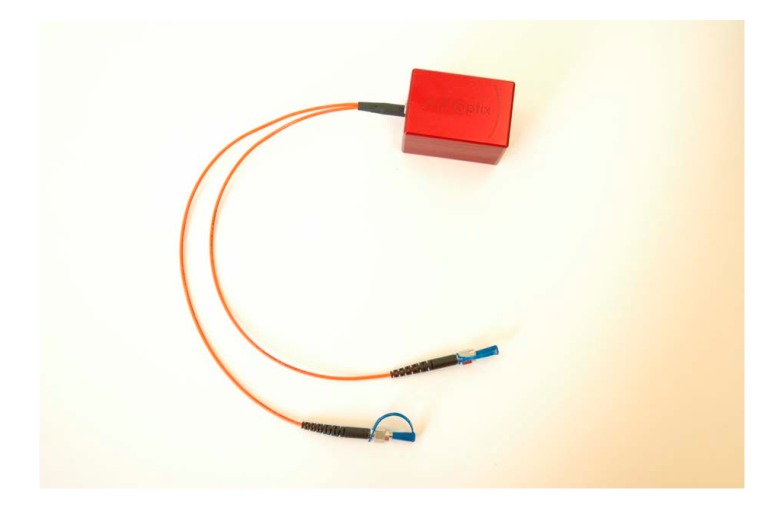
A miniature FTS, including driving electronics, MEMS and fibers. (Reprinted with permission from Reference [29] IEEE).

Based on this actuator, a miniature FTS was designed by Briand et al. in 2007 [29]. The interferometric module involved MEMS, actuation electronics to drive the MEMS and give a feedback of the motion, optical component to bring the light onto the surface of the MEMS mirrors, and two fibers for in-coupling and out-coupling the light (see Figure 6). The instrument was compact and portable, and in principle, easily upgradeable to any wavelength above 2.6 μm up to 5 μm, which depended on the photodetector. In their experiment, a single InGaAs photodiode was applied to record the spectra. This FTS was able to measure 400 spectra per second.

**Figure 7:**
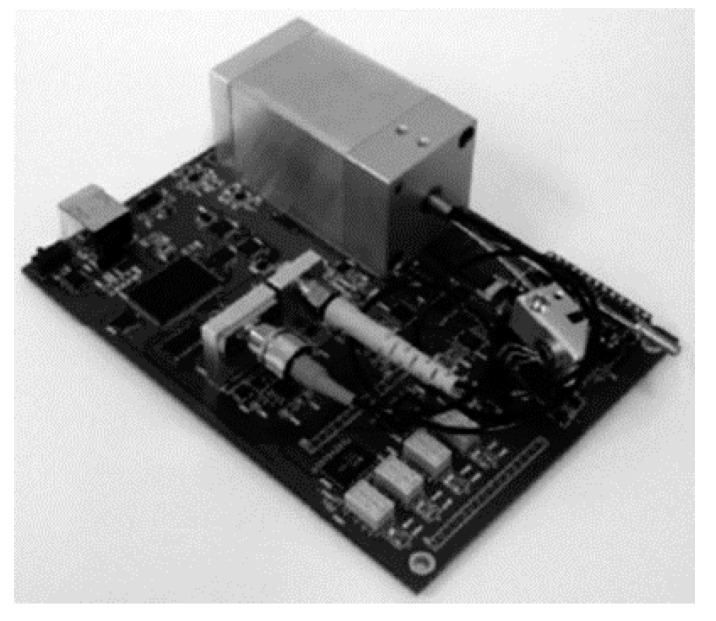
Portable FTS for near-infrared/mid-infrared (ARCspectro ANIR/AMIR) utilizing an in-plane electrostatic MEMS lamellar grating. (Reprinted with permission from Reference [30] SPIE).

Further, FTS systems for near-infrared (ARCspectro ANIR) and mid-infrared (ARCspectro AMIR) were developed based on this type of MEMS by Merenda et al. in 2010 [30]. The new design had a pitch of 100 μm and an 8 mm^2^ active area. This FTS consisted of an interferometer, a VCSEL diode to control the grating movement, and USB communication on a single PCB (see Figure 7). The overall size was 10 cm × 15 cm × 7 cm and the weight was 850 g. It could generate a maximal OPD of more than 1 mm, corresponding to a spectral resolution of fewer than 8 cm^−1^. The wavelength stability was <0.5 nm at 1500 nm at stable conditions.

**Figure 8:**
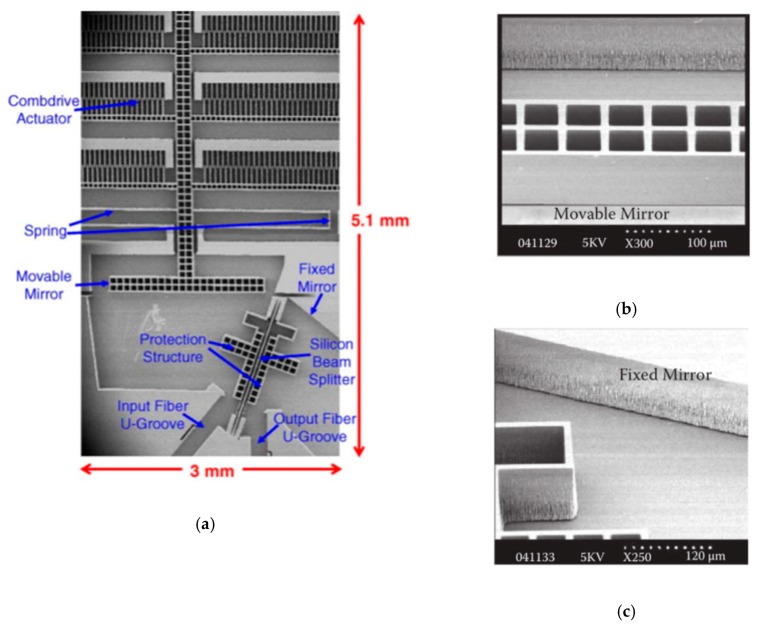
An in-plane electrostatic comb-based FTS: (**a**) SEM of the micromachined FTS; (**b**) SEM of the movable micromirror with the sidewall obtained by potassium hydroxide (KOH) etching; (**c**) SEM of the fixed mirror with the sidewall defined by deep reactive ion etching (DRIE). (Reprinted with permission from Reference [31] Elsevier).

An in-plane integrated FTS microsystem was implemented based on another sidewall electrostatically driven MEMS micromirror by Yu et al. in 2006 [31]. The system integrated a MEMS movable mirror, a fixed mirror, a beam splitter, and fiber U-grooves into a single SOI wafer (see Figure 8a). The U-grooves were used for passive alignment of optical fibers. All the components were made of the (110) silicon device layer of SOI wafers. The fabrication process combined deep reactive ion etching (DRIE) with wet potassium hydroxide KOH etching, realizing a good surface quality of the sidewall. The crystallographic (110) silicon device layer only allows two possible directions, which makes the mirrors oriented at 70.53° not typical 45°. Figure 8b,c show the details of the fixed mirror and the movable mirror, respectively. The fixed mirror had a large surface roughness as it could only be fabricated by DRIE. The device realized in-plane integration of the FTS system, and the overall size was only 4 mm × 8 mm × 0.6 mm, but the area of the comb drive structure occupied about 4 mm × 4 mm. The maximum displacement of the movable mirror was about 25 μm under 150 V at a frequency of 5 Hz. The corresponding maximum OPD was 50 μm, and the spectral resolution measured at a wavelength of 1500 nm was about 45 nm.

**Figure 9:**
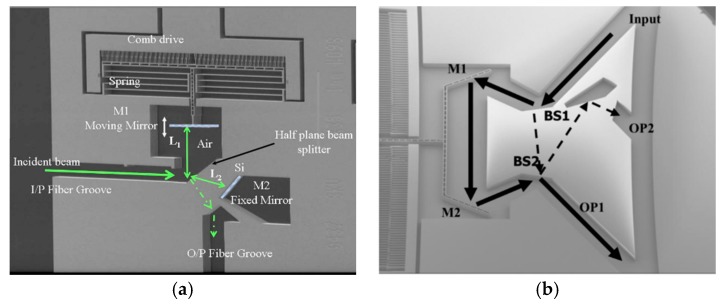
In plane-integrated FTS: (**a**) Classical Michelson interferometer configuration; (**b**) Mach-Zehnder configuration. (Reprinted with permission from Reference [33] SPIE).

Khalil et al. successively proposed two fiber-coupled in-plane integration FTS systems based on the classical Michelson interferometer and Mach-Zehnder interferometer in 2009 and 2011, respectively [32,33]. All of the optical components were located on a single SOI chip. A platform, which was called silicon-integrated micro-optical system technology (SiMOST^TM^, Si-Ware Systems, Cairo, Egypt) [34], was developed and utilized in the manufacturing process. The Michelson interferometer system (see Figure 9a) was realized by applying a special single-medium interface (silicon-air) beam and a vertical comb-based translational mirror. The reflective surfaces of both the moving mirror and fixed mirror could either be metal-coated or an air-silicon Bragg mirror using DIRE. The comb-drive actuator had 126 fingers which could achieve a vertical displacement of 48 μm at resonance. This MEMS chip was mounted on a printed circuit board, and mixed laser sources of 1310 nm and 1550 nm were inserted to the FTS using an optical fiber. 

The Mach-Zehnder interferometer (see Figure 9b) reported by Khalil et al. had two transmission outputs that were utilized to cancel the source fluctuation noise and increase the SNR. Two silicon-air beam splitters and two metallic mirrors were used in the system. The overall size of the chip was only 1 mm × 2 mm. When applying a voltage of 70 V, the micromirror could achieve a maximum OPD displacement of 135 μm at resonance, and the measured spectral resolution of 1550 nm was about 25 nm.

**Figure 10:**
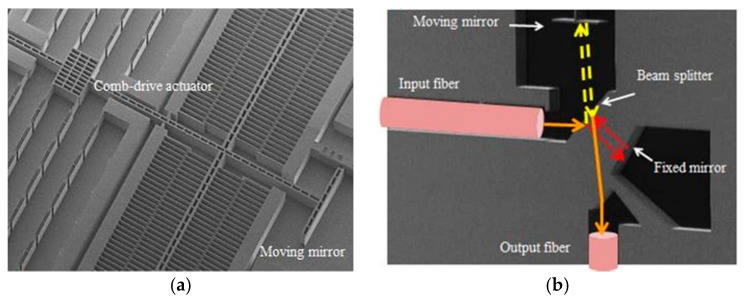
On-chip in-plane electrostatic comb-based FTS: (**a**) Comb-drive actuator; (**b**) optical components. (Reprinted with permission from Reference [35] IEEE).

Mortada et al. presented another on-chip Michelson interferometer based FTS in 2014 [35]. This MEMS actuator (see Figure 10a) was working in a push-pull condition to produce a large displacement. A double folded flexure structure was applied to reduce the parasitic torque usually happened in fabrication tolerance. The whole FTS device comprised a half-plane beam splitter, fixed mirror, moving mirror attached to the MEMS actuator, and fiber grooves employed for optical fiber insertion in an alignment-free manner (see Figure 10b). Etching depth larger than 300 μm was achieved with vertical surface angle and scalloping depth smaller than 0.1° and 60 nm, respectively. To demonstrate the improvement with larger depth, multi-mode optical fibers with core diameters of 62.5 μm and 200 μm were applied to transmit the white light to SOI chips with 90 μm and 200 μm device layer heights. Experimental results showed there was a 12 dB increase in the interference signal, which was a significant boost for the SNR.

**Figure 11:**
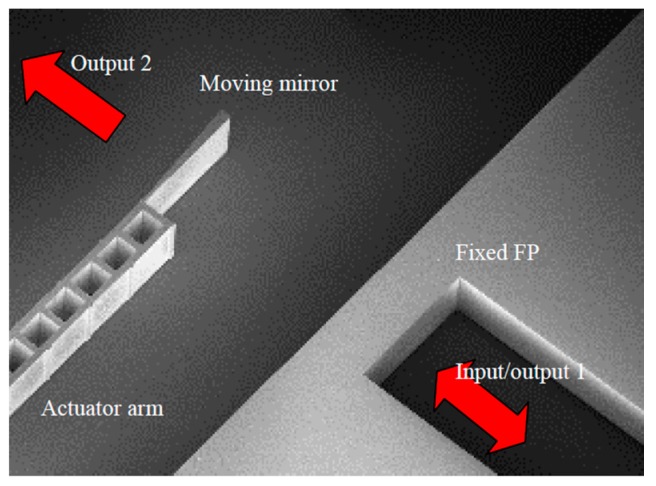
Cascaded FP interferometers based FTS. (Reprinted with permission from Reference [36] SPIE).

Eltagoury et al. developed a cascaded low-finesse Fabry-Perot interferometers based FTS (see Figure 11) in 2016 [36]. Two Fabry-Perot interferometers were applied to replace a traditional Michelson interferometer in this system. A fixed Fabry-Perot interferometer was a silicon block that could generate spectral modulation, and thus, creating a shifted version of the interferogram away from the point of zero spacing between the two mirrors. The interferogram was then produced by the scanning Fabry-Perot interferometer attached to a relatively large-stroke electrostatic comb-drive actuator. This compact device was fabricated on an SOI substrate using DRIE with a simple MEMS process flow. The Fabry-Perot interferometer configuration simply arranged the mirrors in a line, making it much tolerant to the misalignment errors. This cascaded Fabry-Perot interferometer based FTS has been demonstrated reliably through a narrow laser source of 1550 nm and a wide band spectral source composed of a SLED centered around 1300 nm.

#### 3.1.2. Out-of-Plane Electrostatic MEMS Micromirrors and FTS

The in-plane electrostatic MEMS micromirrors have good movement stability without tilting problems. However, the mirror quality is commonly poor and unable to coat gold, aluminum, silicon film which have high reflectance to near infrared light. The FTS system’s luminous flux is limited, due to the height of the mirrors determined by the device layer thickness of the SOI wafer, and the displacement is also restricted. Thus, out-of-plane electrostatic MEMS designs have been explored.

**Figure 12:**
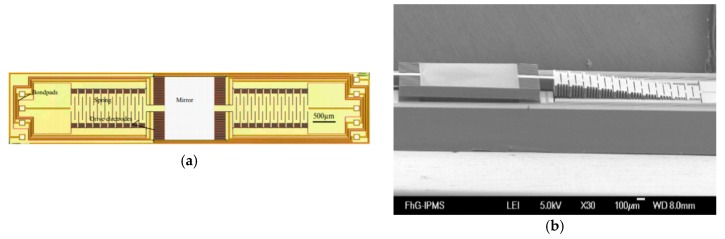
Layout (**a**) and SEM image (**b**) of a type A translational MEMS micromirror, using two long bending springs as mirror suspension. (Reprinted with permission from Reference [37] SPIE).

Sandner et al. designed an out-of-plane moving electrostatic translational MEMS micromirror (type A design) in 2007, which was composed of the central mirror, long cantilever and comb-drive components (see Figure 12) [37]. The actual mirror was suspended on two long bending springs. The chip size was 1.8 mm × 9 mm, and the mirror size was 1.5 mm × 1.1 mm. The mirror was hermetically packaged in a 100 Pa vacuum chamber to reduce the impact of air damping, and thus, achieved a maximum vertical displacement of 200 μm with 40 V driving voltage at 5 kHz resonance. It could produce a spectral resolution of 25 cm^−1^ theoretically.

**Figure 13:**
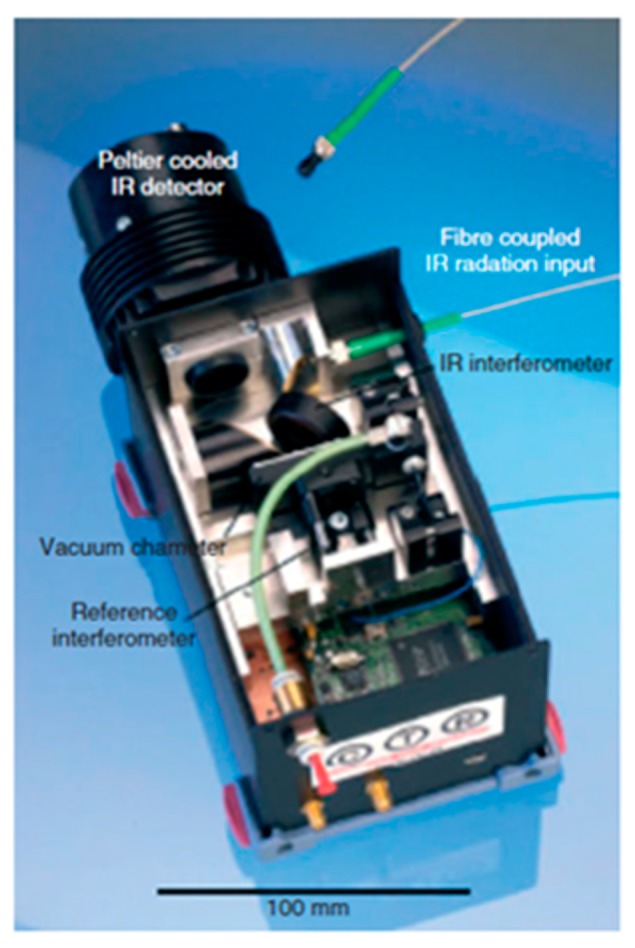
A compact FTS using type A MEMS micromirror. (Reprinted with permission from Reference [37] SPIE).

Type A translational MEMS micromirror was integrated into a small vacuum chamber with optical windows located parallel to the front and back side of this MEMS mirror. A compact FTS was fabricated using type A MEMS micromirror (see Figure 13) in 2007. This MEMS micromirror was operated at 100 Pa in a vacuum chamber. A Peltier cooled IR detector was applied for recording interference signals. A temperature-controlled VCSEL diode monitored the position and velocity of the MEMS mirror in real time to increase the spectral accuracy. This MEMS FTS could produce a resolution of 35 cm^−1^ for a wavelength range of 2.22 μm to 5.55 μm. The measured polystyrene spectrum was consistent with the standard data.

**Figure 14:**
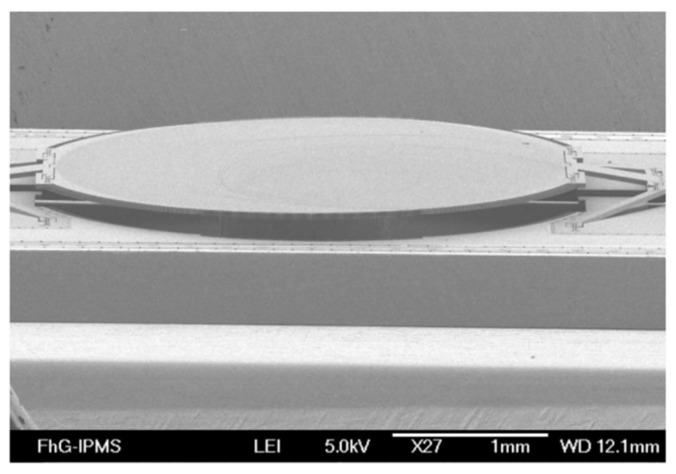
SEM and suspension layout of type B translational MEMS micromirror, using pantograph-type suspensions. (Reprinted with permission from Reference [37] SPIE).

Based on the design of type A translation MEMS micromirror, an improved design of type B, applying two pantograph-like spring mechanisms for mirror suspension was realized (see Figure 14) in 2007. In order to acquire a sufficiently large aperture for optical throughput, circular shape of 3 mm diameter was chosen for this mirror plate. A reduced resonance frequency of 500 Hz was chosen to improve the SNR. This actuator was designed for a stroke of 500 μm, greatly increasing spectral resolution to 10 cm^−1^ theoretically. However, only 280 μm displacement could be measured at a maximum under the influence of superimposed parasitic torsional oscillation modes.

**Figure 15:**
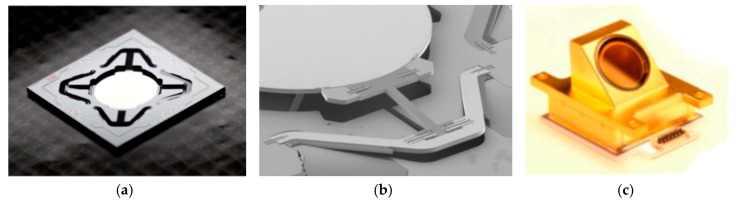
A large displacement vertical electrostatic comb-driven MEMS micromirrors: (**a**) Photograph of new MEMS device at 400 μm mechanical predeflected; (**b**) modified MEMS design with D = 5 mm and a stroke of 1 mm; (**c**) MEMS vacuum package with ZnSe window. (Reprinted with permission from Reference [38] SPIE).

An optimized translator MEMS with a new version of pantograph design was later developed by Sandner et al. in 2014 with a displacement of 1.2 mm at 30 Pa and 50 V environment [38]. It was designed to enable a spectral resolution of 8 cm^−1^ for a wavelength range 2.5 μm to 16 μm at 500 Hz scan rate using a 5 mm diameter round micromirror. The modified translatory MEMS actuator was made up of four pantograph suspensions in the fourfold rotationally symmetric configuration in contrast to two pantographs used for previous type B design (see Figure 15a). It was driven electrostatically at resonance by in-plane vertical comb drives placed at each of the four pantographs to increase the driving efficiency (see Figure 15b). This MEMS device has been encapsulated in a hybrid optical vacuum package (see Figure 15c) to reduce the significant viscous gas damping in normal ambient. While at the normal atmospheric pressure, the driving voltage of 40 V could only produce a displacement of about 160 μm at resonance. The large size of the electrostatic MEMS micromirror could be helpful in improving the displacement output. However, a large mirror of 5 mm produced 200 nm distortion in the driving process, which affected the divergence of the light beam. In addition, the comb structure usually occupied a large space and seriously affected the fill-factor of the micromirror, which also indicated that the displacement generated by the unit drive area was relatively low.

**Figure 16:**
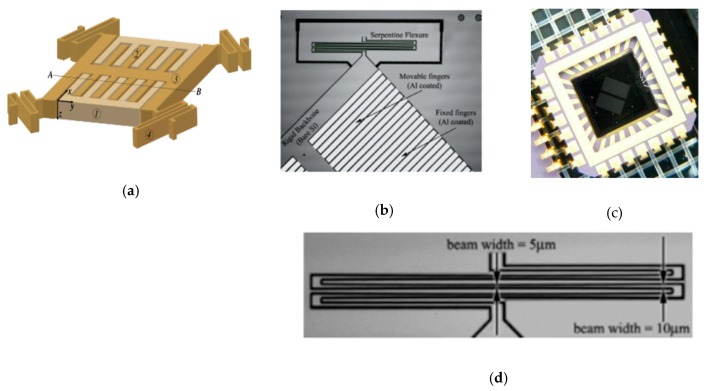
An electrostatic actuator for Lamellar grating based FTS: (**a**) FTS structure schematic: (1) Fixed fingers, (2) Movable fingers, (3) Rigid backbone, (4) Folded flexures; (**b**) fabricated MEMS device; (**c**) packaged device; (**d**) closed-up view of the serpentine flexure. (Reprinted with permission from References [39,40] IOP Publishing, Ltd.).

Ataman et al. reported An out-of-plane vertical comb-drive actuator for Lamellar grating based FTS in 2006 [39,40]. Two sets of electrostatic comb fingers were simultaneously applied as an actuator and a variable depth diffraction grating (see Figure 16a–c). In order to enhance the robustness, the comb fingers were symmetrically placed on a rigid backbone. The length and width of the comb fingers for both static and movable were 1.2 mm and 70 μm, respectively, where the gap between them was 5 μm. A high width-to-gap of the comb fingers realized a high fill factor and well optical efficiency, but the grating period was also increased, which led to smaller separation between diffraction orders. The movable fingers were carried by 250 μm wide H-shaped backbone connected to the fixed frame through four folded flexure beam. The folded flexure structure (see Figure 16d) had uniform stress distribution and could provide low stiffness within a compact structure by varying the cross-sectional width. Only the moving part of the MEMS device was the resonating structure. Out-of-plane resonant mode operation of the comb actuator could generate a peak-to-peak 106 μm deflection in ambient pressure under 28 V square-wave excitation, and could provide a large clear aperture of 3 mm × 3 mm for the incident beam. The Lamellar grating based FTS was constructed by employing the MEMS device, a single detector, and an electronic circuit for processing the detector output. The theoretical spectral resolution of this FTS was 0.4 nm in the visible range and 3.2 nm at telecom wavelengths.

**Figure 17:**
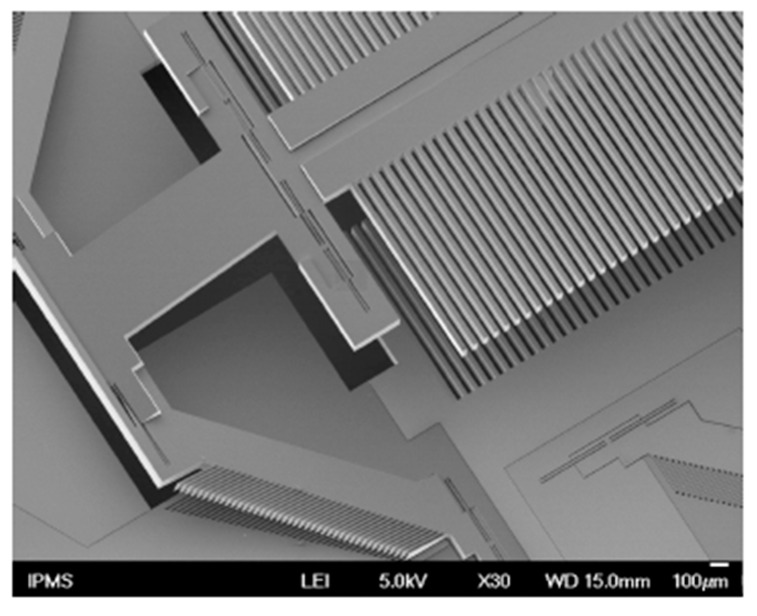
SEM picture of the fabricated deflected device. (Reprinted with permission from Reference [41] IEEE).

Seren et al. implemented another out-of-plane resonant mode electrostatic MEMS for Lamellar grating based FTS in 2010 [41]. The device had a clear aperture of 10 mm × 10 mm. The fabricated MEMS consisted of fixed and movable parallel fingers, which operated as a variable depth grating (see Figure 17). This actuator transferred the motion to micromirror through four pantograph structures. Two comb structures were carried by either side of each pantograph to motivate the device. In addition, the width of fingers and gap between fingers for grating were kept constant. This device was actuated through both comb fingers and grating fingers which were placed at the pantographs to make the electrostatic force maximization. A maximum peak to peak deflection of 355 μm was achieved under 76 V and 971 Hz excitation environment.

#### 3.1.3. Summary

Table 1 summarizes several parameters of electrostatic MEMS micromirrors and FTS. The electrostatic actuators have fast response speed. However, high voltage is required, and hundreds of volts only produce several tens of micrometers. Moreover, high voltage introduces electronics complexity and material compatibility issues. The electrostatic mechanisms commonly operate in resonant frequency and vacuum encapsulation to increase the displacement. On the other hand, pull-in occurs in electrostatic actuators, which may lead to irreversible damages for short circuit, arcing and surface bonding. The pull-in behavior prevents the displacement of an actuator from reaching its full-gap allowable range, so that only 1/3 of the initial gap size could be used.

### 3.2. Electromagnetic MEMS Based FTS

Electromagnetic based MEMS mirrors typically have such characteristics as relatively large displacement, low voltage, and high speed [27]. Magnetization is a phenomenon when a magnetic field causes internal magnetic polarization of a piece of magnetic material within the field. A piece of magnetic material comprises magnetic domains. Each magnetic domain involves a magnetic dipole. The intensity of the internal magnetization of the magnetic material relies on the ordering of these domains. A net internal magnetic field of the magnetic material is produced via these domains, if they are slightly aligned. The magnetic field may come from a permanent magnet, an integrated coil, an external solenoid, or their hybrid manner. Ferromagnetic materials (e.g., iron (Fe), nickel (Ni), or permalloy (Fe-Ni)) are often applied in MEMS actuators [27]. The force-generating structure of an electromagnetic microactuator is commonly placed on a chip, which can be a permanent magnet (hard ferromagnet), a soft ferromagnet, or an integrated coil. When an external magnetic field is present, an internal magnetization is generated in the ferromagnet. Hard ferromagnets can retain certain magnetic polarization when there is no external magnetic driving field, while soft ferromagnets can exhibit internal magnetization only when it is placed in a biasing, external magnetic field. Lorentz-type and magnetic pole type are two kinds of electromagnetic MEMS micromirrors for FTS, which are detailed below.

#### 3.2.1. Lorentz-Type Electromagnetic MEMS Micromirrors and FTS

**Figure 18:**
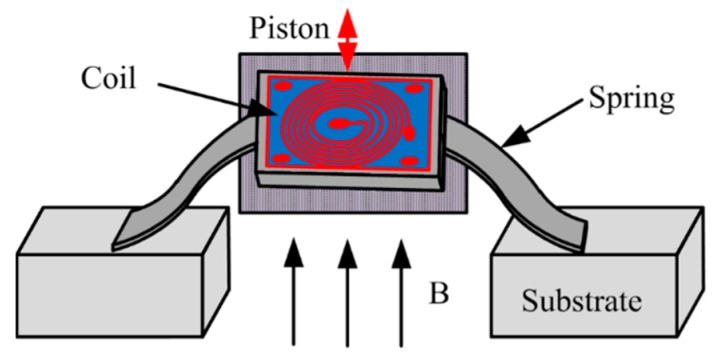
Coil-based magnetic actuators.

When a current is injected into a conductive coil which is perpendicular to an external magnetic field, the coil will experience a magnetic force (Lorenz force). The magnetic force can be applied to produce either in-plane or out-of-plane linear motion. The Lorenz force varies with the input current. Coil-based magnetic actuation can generate either piston motion or rotation depending on the spring design and the external magnetic field direction (see Figure 18).

**Figure 19:**
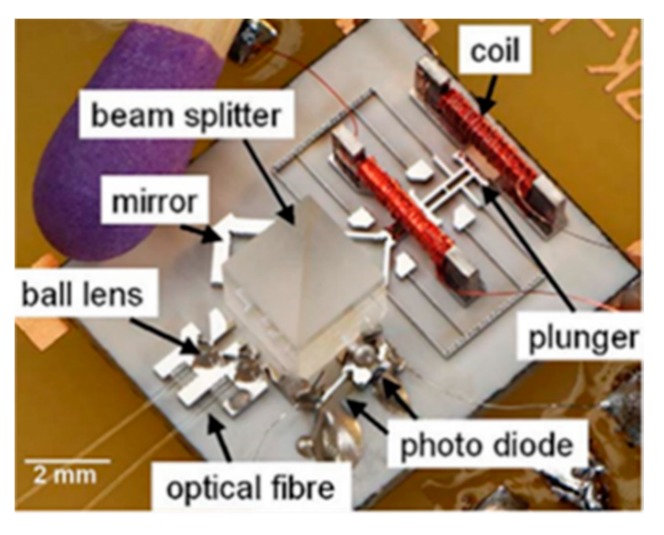
Top view of an electromagnetically actuated miniature FTS using the LIGA process. (Reprinted with permission from Reference [43] Elsevier).

Wallrabe et al. reported an electromagnetic actuator driven large displacement movable micromirror using a LIGA fabrication process in 2005 [42]. This electromagnetic micromirror, a collimator, a photodetector and other optical elements were integrated together on a single substrate (see Figure 19) [8,43], forming a miniature FTS optical platform with a footprint of 11.5 mm × 9.4 mm. The actuator had two assembled microcoils with soft magnetic Fe-Ni alloy cores. The movable plunger was suspended by a set of four folded cantilever beams, and the coils were employed at either end of the plunger. The plunger could be pulled in both directions, thus, doubling the travel range.

The produced magnetic force was related to the number of windings in the coil. With 300 windings, the maximum displacement was about 110 μm at 12 mW. When the input power exceeds 12 mW, the actuator started to exhibit instability, due to the occurrence of pull-in behavior, which greatly limited the useful displacement of the movable mirror. The available displacement of the actuator was only 54 μm, and an 850 nm laser was applied to measure the mirror displacement. The spectral resolution of this FTS was 24.5 nm at 1540 nm. Although this electromagnetic actuator achieved a large scan range and large optical aperture, due to the presence of a pull-in behavior, only a fraction of the full scan range could be used. In addition, the LIGA process is very expensive and difficult to access.

**Figure 20:**
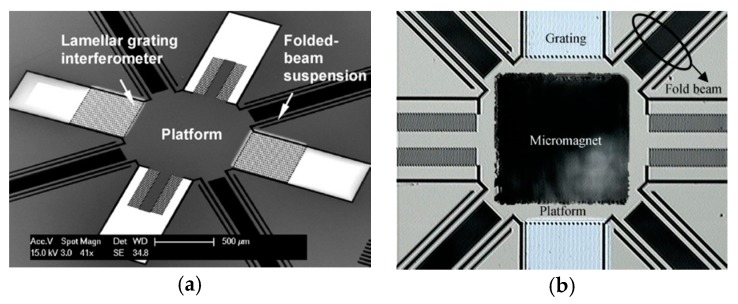
An electromagnetically driven Lamellar grating based FTS: (**a**) SEM; (**b**) microscope image. (Reprinted with permission from Reference [44] IOP Publishing, Ltd.).

An electromagnetically driven Lamellar grating based FTS was developed by Yu et al. in 2008 [44]. The electromagnetic actuator was applied to drive the movable surfaces of the lamellar grating to move bi-directionally (see Figure 20a,b). The size of the permanent magnet was 700 μm × 700 μm × 500 μm, which was placed perpendicular to the central platform. The actuation of this FTS was achieved by a driving coil positioned under the device. When applying 129 mA-amplitude currents to the actuation coil, a large deflection of 125 μm was produced. The resultant force varied linearly with the injecting current, which greatly simplified control procedures. Moreover, this design avoids pull-in instability so that larger deflections (hundreds of microns) can be generated only by increasing the applied current. The measured spectral resolution was 3.8 nm at 632.8 nm and 3.44 nm at 532 nm.

**Figure 21:**
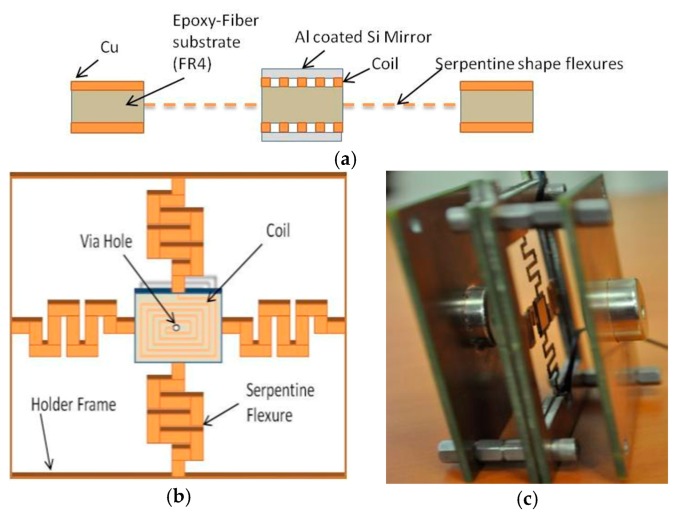
Design of the FR4 Lorentz-type electromagnetic actuator: (**a**) Side view of the system; (**b**) top view of the system; (**c**) fully assembled device. (Reprinted with permission from Reference [45] AMER SOC MECHANICAL ENGINEERS).

Baran et al. proposed an electromagnetically actuated Flame-retardant-4 (FR4) scanner replacing the movable mirror in Michelson interferometer in 2011 [45]. FR4 was a composite material which has been used for PCB with well-engineered electrical, mechanical and thermal properties. The FR4 actuator (see Figure 21a) could enhance the out-of-plane translation when applying two opposing magnets approach to suppress the torsion effects. The central movable part was anchored via four flexures to produce out-of-plane resonance frequency (see Figure 21b). The serpentine shape flexures were used to increase torsion mode stiffness, and decrease out-of-plane mode stiffness. The mirror tilting was suppressed by applying a corner cube reflector. This actuator could generate ± 162.8 μm out-of-plane translation at 149 Hz resonate mode with 120 mVpp applied voltage, leading to a spectral resolution <1 nm. The whole device was 7 cm × 8 cm with a mirror size of 1 cm × 1 cm (see Figure 21c). However, since the FR4 material was extremely soft, the actuator could not stop under such big current and could not come back to the original point, which dramatically decreases the accuracy.

#### 3.2.2. Magnetic Pole-Type Electromagnetic MEMS Micromirors and FTS

**Figure 22:**
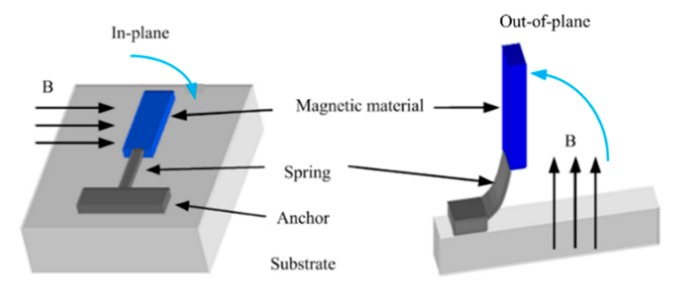
Electromagnetic actuators with magnetic materials formed on the movable structure.

Magnetic pole-type electromagnetic MEMS actuators (see Figure 22) with magnetic materials are deposited on the movable parts. The magnetic poles are generated at the ends of the magnetic material coated movable part when an external magnetic field is applied. An interactive force, either attractive or repulsive force, is then generated between the movable part and the magnetic field. Such actuation can be in-plane or out-of-plane regarding the external field direction.

**Figure 23:**
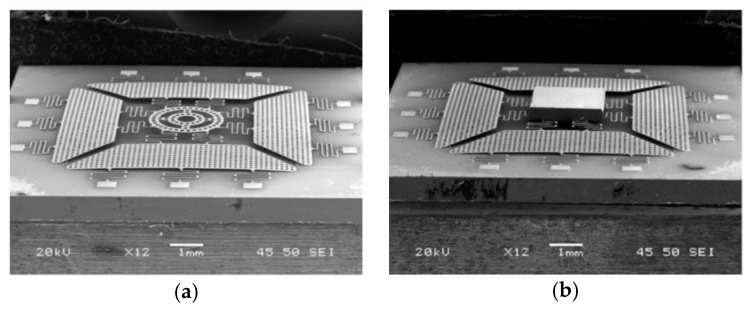
Prototype pf the translation micromirror: (**a**) Nickel film; (**b**) nickel film bonded with the mirror plate. (Reprinted with permission from Reference [46] IOP Publishing, Ltd.).

Xue et al. introduced a large displacement with high surface quality in-plane electromagnetically actuated translation micromirror for FTS in 2016 [46]. The actuator comprised a nickel film (see Figure 23a) fabricated by MetalMUMPs [47], and a solenoid located underneath the film. The nickel film could curve up via the residual stress gradient and a curve-up mechanism, including four trapezoidal plates and anchoring springs. A size of 2 mm × 2 mm mirror plate was connected to the central ring of the nickel film (see Figure 23b). The translation was realized by a solenoid attracting the nickel film along with the mirror plate downwards. A quasi-static displacement of 123 μm was generated at 400 mA. The mirror achieved a high surface quality with 15.6 nm curvature radius and 2 nm surface roughness.

**Figure 24:**
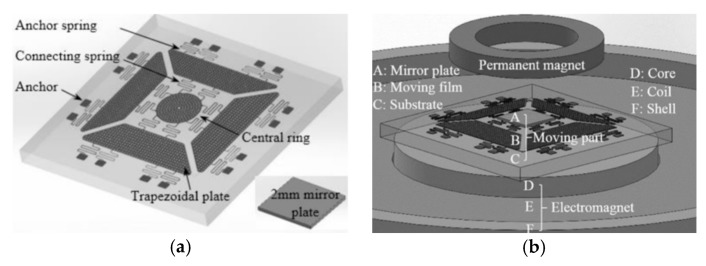
Concept of the micromirror: (**a**) Moving film of the micromirror; (**b**) structure of the micromirror. (Reprinted with permission from Reference [48] IOP Publishing, Ltd.).

A repulsive magnetic force driven out-of-plane micromirror with large displacement and high surface quality was later developed for FTS by Xue et al. in 2017 [48]. This design did not need the residual stress gradients to curl up the moving film. It applied a permanent magnet ring above and an electromagnet underneath the moving film (see Figure 24b) to generate repulsive magnetic force for moving the film to generate out-of-plane motion. The moving film (fabricated by MetalMUMPs [47]) was connected to a 2 mm × 2 mm mirror plate using non-touching bonding technology (see Figure 24a). The micromirror had a radius of curvature of 9.2 m and surface roughness of 12 nm. The maximum displacement of 144 μm was achieved when 140 mA was injected into the electromagnet. However, only 30 μm translation could be used in FTS, due to 0.3° tilting. The measurement accuracy of 2.19 % for a 532 nm laser beam was obtained. 

#### 3.2.3. Summary

Table 2 summarizes several parameters of electromagnetic MEMS micromirrors and FTS. Electromagnetic actuators designed for FTS exhibit larger displacement and lower response speed compared to electrostatic actuators. However, electromagnetic actuators typically have larger device size to hold the external electromagnetic field, thus, reducing its compactness. At the same time, the fabrication may cause asymmetry of the electromagnetic actuation structure, and the magnetic field encapsulation is not precisely coaxial, leading to the tilting of the MEMS micromirror.

### 3.3. Electrothermal Actuation MEMS Based FTS

**Figure 25:**
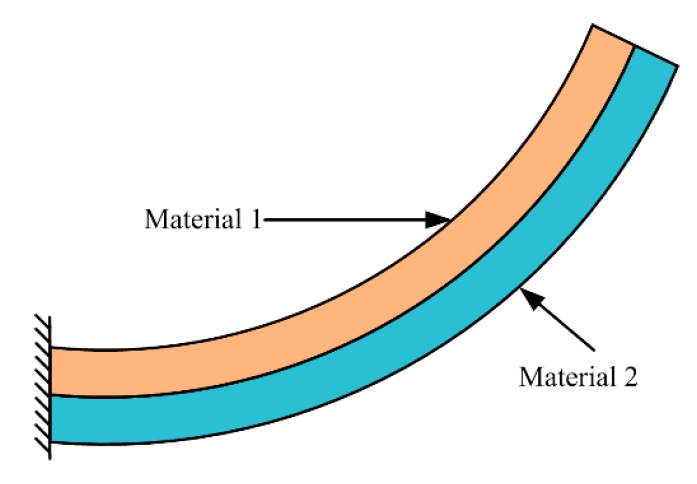
Electrothermal bimorph structure.

Electrothermal actuators are generally based on electrothermal bimorph actuation which is realized by temperature variable via Joule heating induced from injecting an electrical current to a resistive heater embedded in a bimorph beam. A bimorph structure (see Figure 25) comprises two layers of materials with different coefficients of thermal expansion (CTEs). When the temperature changes, thermal stress is generated, due to the difference of the CTEs of the two bimorph layers. The beam curls toward the layer with a lower CTE value when the temperature rises; then, a transverse beam bending is generated [9]. The electrothermal MEMS micromirrors designed for FTS can also produce in-plane and out-of-plane motions, which are described below.

#### 3.3.1. In-Plane Electrothermal MEMS Micromirror and FTS

**Figure 26:**
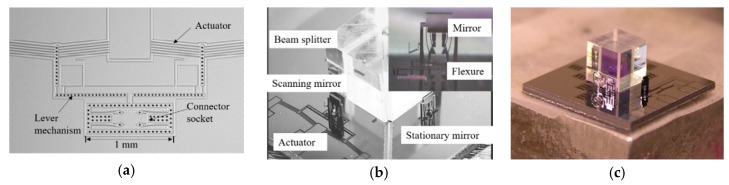
(**a**) An electrothermal scanning mechanism with a lever mechanism; (**b**) an assembled FTS; (**c**) an assembled FTS with a ball lens. (Reprinted with permission from Reference [49] SPIE).

Sin et al. proposed a micro FTS based on in-plane electrothermal actuator in 2006 [49]. All the optical components were fabricated on a micro-optical bench using the DRIE process on an SOI wafer. V-beam shape structures were applied as an electrothermal actuator. The stroke was amplified by a lever mechanism connected to the end of the actuator (see Figure 26a). A symmetric structure of two actuators and two lever mechanisms were employed to enhance the force and eliminate the stage rotation. The sockets were female mechanical flexure structure that enables a precise mirror assembly. The mirror could be picked up by a passive microgripper by inserting the gripper tip into the flexure structure. The overall size of this interference platform was only 10 mm × 10 mm (see Figure 26b), and the area of the micromirror was about 0.5 mm × 0.45 mm. A ball lens was used to collimate incoming light when the micro FTS was combined with a fiber light source (see Figure 26c). The maximum displacement of the movable mirror, driven by a voltage of 22 V was about 30 μm, corresponding to 60 μm OPD. The tilting angles of the mirror were from −2.5° to 0.8° during several experiments. The measured spectral resolution of this micro FTS was about 25 nm at 632 nm. 

**Figure 27:**
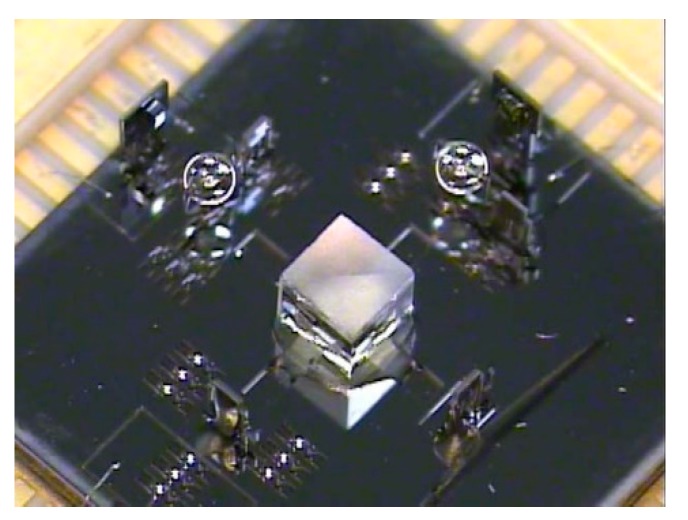
Fully assembled electrothermal actuator-based FTS. (Reprinted with permission from Reference [50] IEEE).

Based on this micro FTS, another in-plane electrothermal actuator based FTS was developed by Das et al. in 2008 [50,51]. The whole device was still fabricated on a 10 mm × 10 mm die. The actuator involved 5 beams of 100 μm × 12 μm × 503 μm, generating a displacement of 45 μm under 45 V driving voltage and the vertical placed assembly gold-coated mirror was 1000 μm × 800 μm in size. The components of this FTS (see Figure 27) were different from the previous design, including two MEMS mirrors, two MEMS holders for ball lenses, two spherical glass lenses, one beam splitter, one single-mode optical fiber, and optionally, one SMT detector and one VCSEL laser source. The assembled FTS was carried out with precision robots using μ^3^ automated micro-assembly station (having 19 precision degrees of freedom). The new design was capable of two FTS instruments: One for the visible range (400–750 nm), the other for near infrared range (700–1800 nm). The calculated spectral resolution of two FTS devices was about 3.6 nm at 600 nm wavelength and 12 nm at 1100 nm wavelength, respectively. 

**Figure 28:**
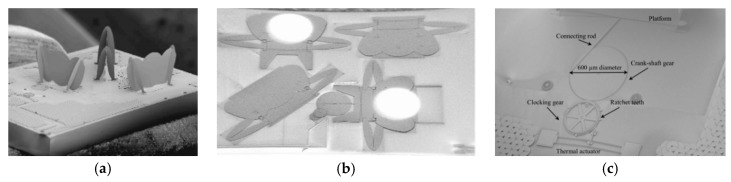
A three-dimensional miniature FTS based on thermal actuator: (**a**) After assembly completion; (**b**) before assembly; (**c**) actuator structure. (Reprinted with permission from References [52,53] SPIE).

Another miniature FTS was realized via in-plane thermal actuator by Reyes et al. (see Figure 28a) in 2008 [52,53]. A special monolithic silicon MEMS solution based on SUMMiT-V technology [54] was used to fabricate the key structures. Compared with the traditional SOI platform having only one device layer, it could make complex multilayer structures of silicon oxide and polysilicon. After the release was completed, the mirror plate was connected to the bottom movable base via a hinge (see Figure 28b). The micromirror was erected by the external force field, and the left and right structures of the mirror plate were raised to form a bracket, thus, locking and keeping the micromirror perpendicular to the base. The movable base was connected to the micro-gear via a connecting rod (see Figure 28c). The transmission structure formed by the micro-gear and the crankshaft was connected to an in-plane thermal actuator via ratchet teeth. The actuator motion could drive the micro-gear to rotate in a specific direction and then drive the connecting rod. A displacement of 600 μm was generated with an open loop stepwise control of the gear system, producing a spectral resolution of 8 cm^−1^. The complete mirror cycle was performed with 225 steps. However, the erection of the components was done manually rather than automatic self-assembly, causing high operating complexity. Moreover, the thin mirror caused mirror warping after metallization, and the curvature was measured at 600 nm. This actuator could only realize stepwise scanning, which limited its application in the short wavelength.

#### 3.3.2. Out-of-Plane Electrothermal MEMS Micromirror and FTS

**Figure 29:**
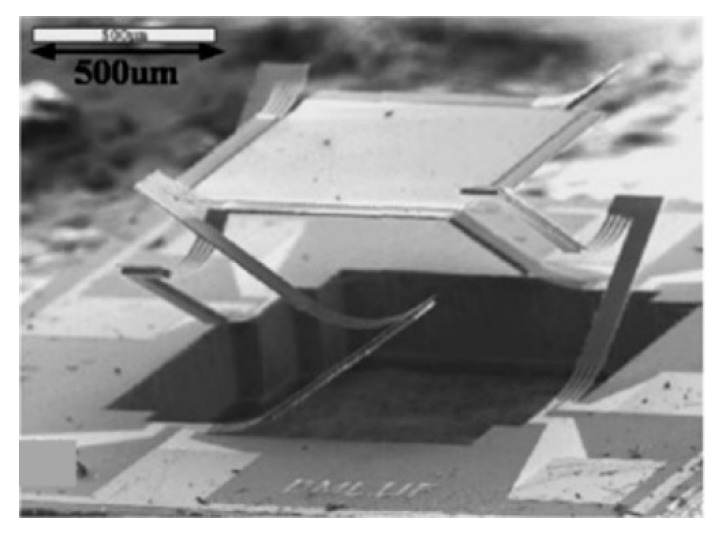
An out-of-plane electrothermal MEMS mirror. (Reprinted with permission from Reference [55] Elsevier).

Since the in-plane electrothermal MEMS micromirrors sustain limited displacement, out-of-plane electrothermal MEMS configurations have been proposed successively. Wu et al. demonstrated a lateral-shift-free (LSF) large-vertical-displacement (LVD) actuator based electrothermal micromirror in 2008 [55] (see Figure 29), whose mirror plate size was 0.8 mm × 0.8 mm. The LSF-LVD actuator comprised two rigid frames and three aluminum (Al)/silicon dioxide (SiO_2_) bimorph beams, which could compensate the lateral shift. It could generate a maximum vertical displacement of 620 μm under 5.3 V driving voltage in an FTS. The scanning tilting of the micromirror was as high as 0.7°, due to the response difference of each driving arm. The displacement was increased to 1 mm in the later experiments [56], but the usable scan range was only 70 μm with tilting <0.06°.

**Figure 30:**
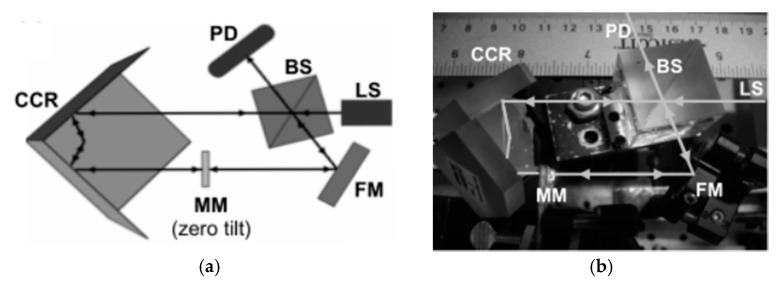
Dual-reflective MEMS mirror-based FTS: (**a**) System Schematic; (**b**) a picture of mirror-tilt-insensitive (MTI)-FTS demonstration setup. CCR: Corner-cube retroreflector. PD: Photodetector. BS: Beam splitter. MM: MEMS micromirror. FM: Fixed mirror. LS: Light source. (Reprinted with permission from Reference [57] IEEE).

A mirror-tilt-insensitive (MTI) FTS based on this MEMS was presented subsequently by Wu et al. in 2009 (see Figure 30a) [57]. It comprised one corner-cube retroreflector and one fixed mirror in each arm of the Michelson interferometer. Both light beams from beam splitter were directed to the dual-reflective MEMS mirror, which could compensate the tilting of the scanning mirror. The actual OPD of this MIT FTS was four times of the physical vertical displacement; thus, the spectral resolution could be enhanced with the increase of the OPD. The whole device size was about 12 cm × 5 cm × 5 cm (see Figure 30b), which could be decreased by reducing the size of optical components. It could achieve spectral resolutions of 19.2 cm^−1^ and 8.1 cm^−1^, which were obtained by the vertical displacement of 131 μm and 308 μm, respectively.

**Figure 31:**
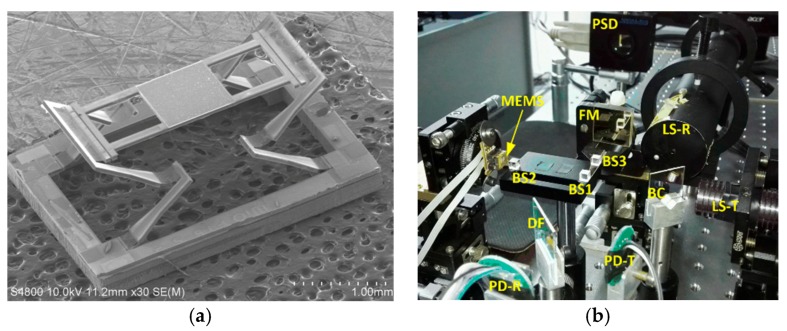
H-shaped LSF electrothermal MEMS based FTS: (**a**) A SEM of MEMS mirror; (**b**) a picture of FTS system. (Reprinted with permission from Reference [58] MDPI).

Wang et al. designed an extended-beam LSF electrothermal MEMS mirror to realize large displacement and small tilting in 2016 (see Figure 31a) [58]. The MEMS device was fabricated using SOI wafers via bulk and surface micromachining. The bimorph was made of Al/SiO_2_, and a platinum (Pt) layer was embedded into the bimorph as a heater. The size of the MEMS device was about 4.3 mm × 3.1 mm, where the mirror plate was about 1.1 mm × 1.1 mm. The maximum vertical displacement could reach 550 μm under 7 V DC driving voltage. The tilt angle was about 0.15° at 3.5 V, which was controlled at ± 0.002° under closed-loop control. The H-shaped electrothermal MEMS mirror based FTS system (see Figure 31b) could generate 860 μm OPD. The measured spectral resolution was about 19.4 cm^−1^.

**Figure 32:**
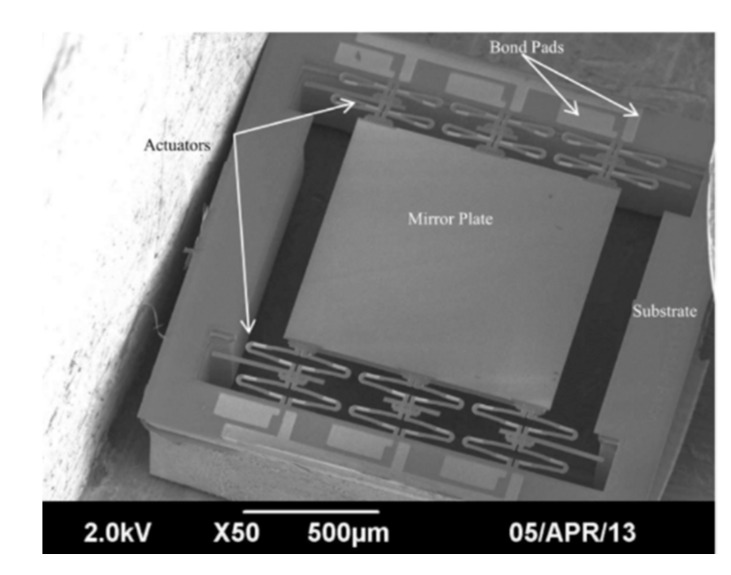
SEM of an actuator array with an elevated mirror plate. (Reprinted with permission from Reference [59] IEEE).

Samuelson et al. reported a folded-dual-S-shaped-bimorph (FDSB) electrothermal micromirror in 2014 [59]. This micromirror had a footprint of 1.9 mm × 1.9 mm with a mirror aperture of 1 mm (see Figure 32). The FDSB design consisted of two separate inverted series connected (ISC) actuators, which were connected by a joint or a hinge. Each S-shaped-bimorph was composed of three segments made of Al/SiO_2_, SiO_2_/Al/SiO_2_, and SiO_2_/Al, respectively. A Pt layer was embedded into the bimorph as a heater. Six pairs of FDSB actuators were placed on the opposing sides of the central mirror plate. The multi-actuators were applied to compensate for the response difference of each actuator and decrease the scan tilting angle. The FDSB micromirror was fabricated on SOI wafers via a combined surface and bulk micromachining process. The maximum displacement of 90 μm was generated with <0.25° tilt at 1.2 V DC.

**Figure 33:**
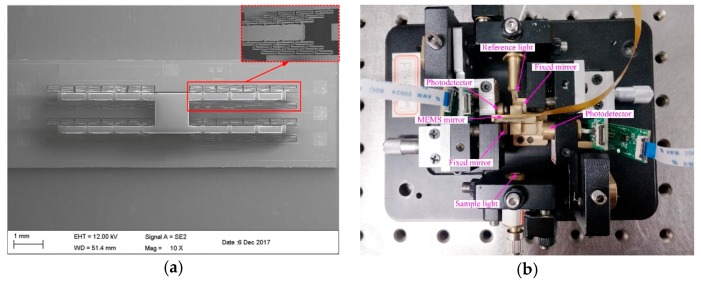
The scan stability H-shaped electrothermal MEMS based FTS: (**a**) MEMS mirror; (**b**) FTS system. (Reprinted with permission from Reference [9] IOP Publishing, Ltd.).

Chai et al. presented a portable FTS using an H-shaped electrothermal MEMS mirror [9]. The mirror plate was connected to a unique H-shaped frame supported by symmetrically distributed thirty-two pairs of innovative three-level-ladder FDSB actuators (see Figure 33a). Each bimorph was S-shaped, including three segments: Al/SiO_2_ film layer, SiO_2_/Al/SiO_2_ film layer, and SiO_2_/Al film layer. titanium (Ti) layer was embedded into the S-shaped bimorph actuator as a resistor. The MEMS mirror (Entire chip: 3.65 mm × 11.4 mm; Mirror plate: 1.4 mm × 1.2 mm) could generate about 200 μm displacement at 5 Hz with only 5 Vpp and maintain a very small tilting of 0.029° without using any complex compensation or closed-loop control. An InGaAs photodetector was applied to record the interferogram, and a 1310 nm laser was used to monitor the MEMS motion. This FTS could cover a wide spectral range of 1000–2500 nm. A spectral resolution of 64.1 cm^−1^ was achieved for this MEMS FTS (see Figure 33b).

#### 3.3.3. Summary

Table 3 summarizes several parameters of electrothermal MEMS micromirrors and FTS. The electrothermal actuators have a relatively large range of movement, compared to electrostatic actuators and electromagnetic actuators. For comparable displacement, much smaller footprints are used in electrothermal actuators. On the other hand, electrothermal actuators require moderate power as electrical current is applied to generate Joule heating, and they have relatively low response speed since the time constant is controlled by thermal heating and dissipation.

### 3.4. Summary of Electrostatic, Electromagnetic, and Electrothermal Actuators for MEMS FTS

As discussed in this section, electrostatic, electromagnetic, and electrothermal actuators have been applied in the miniaturization of FTS devices. Relative advantages and disadvantages of an electrostatic actuator, electromagnetic actuator, and electrothermal actuator are summarized in Table 4.

Electrostatic actuators have high speed and low power consumption, but it is difficult to obtain large scan displacement. Even though there are reports about large piston displacement with electrostatic actuation, those designs require vacuum packaging and resonance operation, which dramatically increase the cost and overall device size. Electromagnetic actuators usually need an external magnetic field, which is not conducive to system integration and may cause electromagnetic interference problems. Electrothermal actuators can reach about 1 mm scan range at a low voltage without the need of a resonant operation, which is highly preferred for FTS applications. Promising results have been achieved from electrothermally-actuated MEMS FTS. 

For an FTS application, the accuracy and repeatability of the acquired spectra are of significance too, which will have implications on the stability of the response and transferability of the calibration models between instruments.

## 4. Conclusions

MEMS presents a huge potential in the miniaturization of FTS devices. With the markets of MEMS based FTS growing rapidly, more demands for new applications are continually emerging. A majority of MEMS based devices have become commercial products. In this article, we have summarized MEMS based FTS systems for spectral analysis and material detection. The basics and common applications of FTS are presented. Three types of MEMS FTS, including electrostatic, electromagnetic and electrothermal, are introduced and shown with examples. The spectral resolution is a key parameter used to evaluate the performance of a MEMS FTS. Besides that, the repeatability and reliability are also two main issues for the commercialization of MEMS FTS to be further mature.

## Figures and Tables

**Table 1:** Comparison of electrostatic micro-electromechanical system (MEMS) micromirrors and Fourier transform spectrometers (FTS).

Authors	Institution	Actuation Type	Core of FTS	Displacement	Work Condition	Device Size	Reference
Manzardo et al.	UniNE	In-plane	MI	77 μm	10 V–amplitude	MEMS chip: 5 × 4 mm^2^ (Mirror: 75 × 500 μm^2^)	[7]
Manzardo et al.	UniNE	In-plane	LGI	145 μm	65 V	MEMS chip: 5 × 5 mm^2^	[19]
Merenda et al.	ARCoptix and EPFL	In-plane	LGI	>500 μm	−	Entire FTS: 10 × 15 × 7 cm^3^	[30]
Yu et al	SNU, Stanford, and SNL	In-plane	MI	25 μm	150 V @ 5 Hz	Entire FTS: 4 × 8 × 0.6 mm^3^	[31]
Khalil et al.	ASU and SWS	In-plane	MI	48 μm	@ resonance	-	[32,33]
Khalil et al.	ASU and SWS	In-plane	MZI	62.5 μm	70 V @ resonance	Entire FTS: 1 × 2 mm^2^	[33]
Mortada et al.	SWS, EP, and ASU	In-plane	MI	62.5 μm or 200 μm	−	−	[35]
Eltagoury et al.	ASU and SWS	In-plane	FPI	−	−	−	[36]
Sandner et al.	IPMS	Out-of-plane	−	200 μm	40 V @ 100 Pa vacuum, 5 kHz	MEMS chip: 1.8 × 9 mm^2^ (Mirror: 1.5 × 1.1 mm^2^)	[37]
Sandner et al.	IPMS	Out-of-plane	−	500 μm	500 Hz	Aperture: 3 mm in diameter	[37]
Sandner et al.	IPMS	Out-of-plane	−	1.2 mm	50 V @ 30 Pa vacuum, 500 Hz	Aperture: 5 mm in diameter	[38]
Ataman et al.	KU and IPMS	Out-of-plane	LGI	106 μm	28 V @ resonance	Aperture: 3 × 3 mm^2^	[39,40]
Seren et al.	KU and IPMS	Out-of-plane	LGI	355 μm	76 V @ 971 Hz	Aperture: 10 × 10 mm^2^	[41]

(UniNE: University of Neuchâtel; EPFL: Ecole Polytechnique Federale de Lausanne; SNU: Seoul National University; SNL: Sandia National Lab; ASU: Ain-Shams University; SWS: Si-Ware Systems; EP: ESIEE Paris; IPMS: Fraunhofer Institute for Photonic Microsystems; KU: Koc University; MI: Michelson Interferometer; LGI: Lamellar Grating Interferometer; MZI: Mach-Zehnder Interferometer; FPI: Fabry-Perot Interferometer).

**Table 2:** Comparison of electromagnetic MEMS micromirrors and FTS.

Authors	Institution	Actuation Type	Core of FTS	Displacement	Work Condition	Device Size	Reference
Wallrabe et al.	Uni Freiburg and FK	Lorentz-type	MI	110 μm	12 mW	Entire FTS: 11.5 × 9.4 mm^2^	[8,43]
Yu et al.	NUS and DSI	Lorentz-type	LGI	125 μm	129 mA–amplitude	−	[44]
Baran et al.	KU	Lorentz-type	MI	325.6 μm	120 mVpp @ 149 Hz	MEMS chip: 7 × 8 cm^2^ (Mirror: 1 × 1 cm^2^)	[45]
Xue et al.	RU	Magnetic pole-type	−	123 μm	400 mA.	Mirror: 2 × 2 mm^2^	[46]
Xue et al.	RU	Magnetic pole-type	−	144 μm	140 mA	Mirror: 2 × 2 mm^2^	[48]

(Uni Freiburg: University of Freiburg; FK: Forschungszentrum Karlsruhe; NUS: National University of Singapore; DSI: Data Storage Institute of Singapore; KU: Koc University; RU: Ryerson University).

**Table 3:** Comparison of electrothermal MEMS micromirrors and FTS.

Authors	Institution	Actuation Type	Core of FTS	Displacement	Work Condition	Device Size	Reference
Sin et al.	UTA	In-plane	MI	30 μm	22 V	Entire FTS: 10 × 10 mm^2^ (Mirror: 0.5 × 0.45 mm^2^)	[49]
Das et al.	UTA	In-plane	MI	45 μm	45 V	Entire FTS: 10 × 10 mm^2^ (Mirror: 1 × 0.8 mm^2^)	[50,51]
Reyes et al.	BML	In-plane	MI	600 nm	−	−	[52,53]
Wu et al.	UF	Out-of-plane	−	620 μm	5.3 V.	−	[55]
Wu et al.	UF	Out-of-plane	MI	131 μm and 308 μm	−	Entire FTS: 12 × 5 × 5 cm^3^	[57]
Wang et al.	SJTU and UF	Out-of-plane	MI	550 μm	7 V DC	MEMS chip: 4.3 × 3.1 mm^2^ (Mirror: 1.1 × 1.1 mm^2^)	[58]
Samuelson et al.	UF	Out-of-plane	−	90 μm	1.2 V DC	MEMS chip: 1.9 × 1.9 mm^2^ (Mirror Aperture: 1mm)	[59]
Chai et al.	USST, WiO Tech, and UF	Out-of-plane	MI	200 μm	5 Vpp @ 5 Hz	MEMS chip: 3.65 × 11.4 mm^2^ (Mirror: 1.4 × 1.2 mm^2^)	[9]

(UTA: The University of Texas at Arlington; BML: Block MEMS LCC; UF: University of Florida; SJTU: Shanghai Jiao Tong University; USST: The University of Shanghai for Science and Technology; WiO Tech: Wuxi WiO Technologies Co., Ltd.).

**Table 4:** Comparison of various actuators.

Various Actuators	Advantages	Disadvantages
Electrostatic actuators	Fast response	Vacuum package and resonance operation
	Low power consumption	Limited displacement
		Pull-in behavior
Electromagnetic actuators	Moderately large displacement	External magnetic field needed
		Large size
Electrothermal actuators	Large displacement	Large power consumption
	Moderately fast response	Sensitivity to environmental temperature changes
